# Predictive Value of Collagen Biomarkers in Advanced Chronic Kidney Disease Patients

**DOI:** 10.3390/biom13020389

**Published:** 2023-02-18

**Authors:** Carina Ureche, Gianina Dodi, Adela Mihaela Șerban, Andreea Simona Covic, Luminița Voroneanu, Simona Hogaș, Radu Andy Sascău, Cristian Stătescu, Adrian Covic

**Affiliations:** 1 Department of Internal Medicine, Faculty of Medicine, “Grigore T Popa” University of Medicine and Pharmacy, University Street nr 16, 700115 Iasi, Romania; 2 Prof. Dr. George I.M. Georgescu Institute of Cardiovascular Diseases, 700503 Iasi, Romania; 3 Biomedical Sciences Department, Faculty of Medical Bioengineering and Advanced Research and Development Center for Experimental Medicine, Grigore T. Popa University of Medicine and Pharmacy of Iasi, 9-13 Kogalniceanu Street, 700454 Iasi, Romania; 4 Niculae Stancioiu Heart Institute, Iuliu Hatieganu University of Medicine and Pharmacy, 400012 Cluj-Napoca, Romania; 5 Nephrology Clinic, Dialysis, Renal Transplant Center, .I. Parhon University Hospital, 700503 Iasi, Romania; 6 Academy of Romanian Scientists, 050044 Bucharest, Romania; 7 Academy of Medical Sciences, 020125 Bucharest, Romania

**Keywords:** procollagen type I carboxy-terminal propeptide, procollagen type III N-terminal peptide, chronic kidney disease

## Abstract

Patients with chronic kidney disease have an increased risk of all-cause death. The value of collagen biomarkers such as procollagen type I carboxy-terminal propeptide (PICP) and procollagen type III N-terminal peptide (P3NP), in end-stage renal disease (ESRD), has not yet been defined (in the literature and in clinics). The purpose of this study was to determine the potential value of these new biomarkers in the prediction of mortality in this population. Plasma PICP and P3NP levels were determined in 140 patients with ESRD, not yet on dialysis, who were followed up for 36 ± 5.3 months. During follow-up, 58 deaths were recorded (41.4%), with the majority of them being cardiovascular deaths (43, 74.13%). Using the ROC curve, the cut-off value for the prediction of mortality for PICP was 297.31 µg/L, while for P3NP, the cut-off value was 126.67 µg/L. In univariate analysis, a value of PICP above the cut-off point was associated with a fivefold increased risk of mortality (hazard ratio (HR) 5.071, 95% confidence interval 1.935–13.29, *p* = 0.001) and a value of P3NP above the cut-off point was associated with a twofold increased risk of mortality (HR 2.089, 95% CI 1.044–4.178, *p* = 0.002). In a multivariable Cox proportional hazards model, PICP values remained independent predictors of mortality (HR 1.22, 95% CI 1.1–1.31, *p* < 0.0001). Our data suggest that the collagen biomarker PICP is an independent predictor of mortality in ESRD patients who are not yet on dialysis.

## 1. Introduction

Chronic kidney disease (CKD) is a major health issue worldwide, with an increasing prevalence, estimated at 9.1% globally [1]. Alongside a high risk for renal complications and disease progression, these patients exhibit an increased risk of all-cause mortality, up to 2.5-fold higher when compared with the general population [2]. In 2017, CKD was responsible for 2.6 million deaths worldwide, with more than half being labelled as cardiovascular deaths (1.4 million) [1]. This phenomenon leads to a continuous process of refining the risk stratification in these patients, aiming towards the early identification of those at high risk, which could allow a personalized intervention. Several biomarkers were already proven useful in this context, such as the classic cardiac troponins, natriuretic peptides, and C reactive protein, or the novel soluble suppression of tumorigenesis-2, Galectin 3, or copeptin [3,4,5,6,7,8,9].

Collagen type I and III are the two major proteins involved in extracellular matrix (ECM) remodelling, which leads to the accumulation of fibrotic tissue in several organs, such as the heart or the kidneys [10]. Although normal wound healing and fibrosis share some similarities, pathological fibrosis leads to remodelling, organ dysfunction, and high mortality [11]. In CKD, ECM remodelling is responsible for podocyte dysfunction and fibrosis, which is a multifactorial process, triggered by chronic inflammation, activation of the renin-angiotensin system, hyperparathyroidism, and a high serum level of fibroblast growth factor 23 (FGF 23), with abnormal calcium, phosphorus, and vitamin D metabolism [12]. Diabetes mellitus is an important cause of CKD and its coexistence can contribute to this process, secondary to oxidative stress and the accumulation of advanced glycation end products (AGEs) [13]. All of these pathways promote accelerated myocardial fibrosis, which is responsible for the early cardiac dysfunction in patients with CKD, secondary to an increase in fibrotic tissue and a decrease in normal functioning cardiomyocytes [14]. This phenomenon translates into the clinical practice, contributing to the high burden of cardiovascular death in this population [2].

The gold standard for quantifying organ fibrosis is a biopsy, but this is an invasive method, which is not available in many centres. The idea that one could identify biomarkers derived from the collagen pathways that could predict the level of organ fibrosis seemed promising.

Fibrillar collagen type I and III are two of the main peptides involved in ECM remodelling. They are synthetized by fibroblasts as promolecules and, for activation, the intervention of two peptidases is required. This leads to the formation of procollagen type I carboxy-terminal propeptide (PICP) and procollagen type III N-terminal peptide (P3NP), respectively [10].

So far, PICP and P3NP have been studied as potential biomarkers for the entire spectrum of cardiovascular disease, ranging from atrial fibrillation and hypertension to cardiomyopathies and heart failure [15,16]. These observations were also confirmed in patients with CKD and hemodialysis, where PICP was correlated with the presence of diastolic dysfunction, mean E/e’ ratio, and increased filling pressures, as a possible phenotype of malignant myocardial fibrosis [17,18]. Additionally, in dialysis patients, an increase in both PICP and Galectin-3 was associated with an increased risk of cardiovascular death [19].

Circulating P3NP, on the other hand, despite promising data in cardiac diseases, did not confirm its potential role as a ‘renal’ biomarker [20]. Still, there are recent data pointing to a potential value for urinary P3NP in patients with CKD [21]. PRO-C3, another molecule involved in the anabolism of collagen type III, which differs slightly from P3NP, was shown to be independently associated with progression and mortality in patients with CKD, suggesting that this pathway, although poorly studied, could be involved in this conundrum [22].

In this context, we performed a cross-sectional prospective observational study, in a significant cohort of patients with end stage renal disease (ESRD), not yet on dialysis, in order to investigate the predictive value of PICP and P3NP for mortality in this population.

## 2. Materials and Methods

### 2.1. Study Population

A prevalent cross-sectional cohort approach was used and patients with ESRD who were not yet on dialysis were assessed for eligibility for inclusion. We included patients who were hemodynamically stable, in sinus rhythm, asymptomatic, and with a glomerular filtration rate (GFR) ≤ 15 mL/min/1.73 m^2^. The exclusion criteria were the presence of a poor acoustic window, a cardiac pacemaker, atrial fibrillation, symptoms of severe heart failure (NYHA class III or IV), severe pulmonary hypertension, active malignancy, and an indication for emergency hemodialysis or a life expectancy of less than one year. All patients signed informed consent forms approved by the Ethics Committee of the University of Medicine and Pharmacy Grigore T. Popa Iași (no.) and of the Clinical Hospital Dr. C. I. Parhon from Iași, Romania (no.). The study can be found on the ClinicalTrials.gov database with NCT05685823 identifier.

### 2.2. Study Design

This study was a cross-sectional cohort study, which involved the screening of 187 patients. At baseline, 140 individuals were included and followed up for three years, as an extension cohort study. After obtaining the informed consent, the patients were examined clinically and with transthoracic echocardiography. The participation in the study involved access to the patients’ medical history and treatment. A venous blood sample was collected and preserved at −80 °C. There were no interferences to be corrected related to the medication or hydration of the patients during the study. The presence or absence of a pre-emptively performed arterio-venous fistula was not considered an exclusion criterion.

The aim of this study was to investigate the predictive value of collagen biomarkers PICP and P3NP in this population, after three years of follow-up. The primary end-point was all-cause mortality. Patients were censored at last follow-up (October 2022).

### 2.3. Demographic, Biochemical, and Echocardiography Parameters

Several demographic and laboratory parameters were recorded at baseline: age, gender, chronic kidney disease vintage (months), creatinine, estimated GFR, diabetes, hypertension, smoking status, obesity and body mass index, ischemic cardiomyopathy, heart failure (NYHA class) hemoglobin, and uric acid. All laboratory tests were performed in the hospital laboratory, by certified staff and with standard procedures.

In order to measure the serum level of PICP and P3NP, enzyme-linked immunosorbent assay (ELISA) was used. Peripheral blood samples were collected in appropriate tubes during the clinical examination. Immediately after coagulation, the serum was separated by centrifugation (10 min. at 4500 rpm) and stored in aliquots in 2 mL Eppendorf tubes at −80 °C for subsequent ELISA. The tubes were marked with a specific ID.

Aliquots of serum samples were thawed naturally at room temperature for each assay. The level of PICP (cat. no. MBS026891) and P3NP (cat. no. MBS045955), purchased from MyBioSource Inc., San Diego, CA, USA, were evaluated according to the manufacturer’s recommendations. Briefly, 50 or 100 µL of standards, blank, and samples were added into appropriate wells in duplicate, followed by 100 or 50 µL of HRP-conjugate reagent and incubation at 37 °C for 1 h. The plate was then washed 4–5 times using a multi channel pipette, and 50 µL of substrate A and 50 µL of substrate B were added to all wells. After incubation at 37 °C for 15–25 min, 50 µL of stop solution was added to each well, and the optical density was determined at 450 nm using the Biochrom EZ Read 400 Microplate Reader (Biochrom, Cambridge, UK). The data were generated in electronic format with the help of the Galapagos Expert Software (Biochrom, Cambridge, UK). All measurements were carried out in triplicate.

Subsequently, the results were calculated using the standard curve constructed by plotting the average absorbance of standards against the known concentrations of standards (25–800 ng/mL for PICP and 3.12–100 ng/mL for P3NP) using Microsoft Excel software.

All echocardiographic evaluations were performed at inclusion, using the Philips CX50 ultrasound system (Andover, MA, USA), with the help of QLAB 7.1 software (Andover, MA, USA). In order to minimize the variability, all ultrasounds were performed by the same observer, according to the recommendations of the American Society of Echocardiography and the European Association of Cardiovascular Imaging [23]. Later, to ensure an unbiased outcome, one other investigator re-examined and interpreted the offline images. All patients benefited from a comprehensive echocardiographic evaluation, of both the cardiac anatomy (diameters, volumes, and mass) and the functionality—the systolic and diastolic function of the left and right ventricles, atrial function, valvular function, and pulmonary artery pressure. As representative parameters, we chose the ejection fraction (EF) and the global longitudinal strain (GLS) for assessing the systolic function of the left ventricle and the mean E/e’ and left atrial volume indexed (LAVI) for the diastolic function of the left ventricle. The GLS was calculated after obtaining three standard views (apical four chambers, apical two chambers, and apical three chambers), with the help of the QLAB 7.1 software (Andover, MA, USA). Cine loops with good image quality and a high frame rate were used. After manually tracing three points of interest in the end diastole, the software automatically traced the endocardial contour, which was corrected as needed. The region of interest (ROI) was automatically estimated and manually adjusted to be adapted to the left ventricular wall thickness. Subsequently, the global longitudinal strain was calculated, with the generation of the bulls eye plot. For this study, we used the mean value reported by the two observers. A normal GLS value was considered to be >−18.9%, adapted to the vendor used [23].

### 2.4. Statistical Analysis

Statistical analysis was performed using SPSS Statistics 25 software (IBM, Armonk, NY, USA). Non-normally distributed variables were expressed as median with interquartile range (IQR), and normally distributed variables as mean ± standard deviation (SD). For categorical variables, Fisher’s exact test was used. An independent *t*-test was used for comparing the means of two independent groups of continuous variables. In order to establish the cut-off for the three biomarkers, the method described by Contal et al. was used [24]. Then, using this established cut-off, Kaplan–Meier curves with log rank and Cox regression analysis were generated in order to determine the value of these biomarkers for predicting mortality. In order to determine the correlations between the variables, Pearson’s correlation coefficient was used. Stepwise multivariate regression analysis including all univariate associates (*p* < 0.05) was used to assess the independent associations between variables.

## 3. Results

### 3.1. Baseline Characteristics

Baseline demographic, biochemical, and echocardiographic characteristics of the study population are depicted in Table 1. After screening 187 patients, a total of 140 patients were included in this study and followed up for three years. The mean age was 59 ± 15 years, with 78 men (55.7%) and 63 females (44.3%) included. The mean creatinine at inclusion was 6.6 ± 2.4 mg/dL, with a mean estimated GFR of 8.7 ± 3.3 mL/min/1.73 m^2^. All patients were hypertensive, 25.7% were obese (mean BMI 26.04 ± 5), 16.4% were active smokers, 11.4% had a history of ischemic heart disease, and 33.5% were diabetics. Furthermore, 52.1% of the patients were in NYHA class I, 47.9% of the patients in NYHA class II, and the mean NT pro BNP level was 250 ± 56 pg/mL. The mean hemoglobin level was 9.72 ± 2 g/dL and the mean uric acid was 7.51 ± 2 mg/dL.

The mean PICP level was 457.2 ± 240 µg/L and the mean P3NP level was 242 ± 199.9 µg/L.

The mean ejection fraction was 53.63 ± 8%, the mean GLS was −10.2% ± 5.3%, the mean E/e’ ratio was 9.8 ± 4.3, and the mean LAVI was 45.8 ± 14.2 mL/m^2^.

### 3.2. Survival Analysis

Patients were followed up for three years. Fifty-eight deaths (41.4%) were recorded and 5 patients were lost to follow-up. The majority (43 deaths, 74.13%) were due to cardiovascular causes, 11 deaths were due to sepsis (18.9%), 2 deaths were due to malignancies (3.4%), and 2 deaths were due to respiratory tract infections (3.4%).

When compared with the group that survived at three years, the group that died had a higher mean age (67.47 ± 9.73 years vs. 53.4 ± 15.9 years, *p* < 0.0001), more severe hypertension (1.15% Grade 2 and 98.85% Grade 3 vs. 13% Grade 2 and 87% Grade 3, *p* = 0.01) and heart failure (58.7% NYHA Class II and 41.3% NYHA Class I vs. 40% NYHA Class II and 60% NYHA Class I, *p* = 0.038), more diabetes (*p* < 0.0001), a lower BMI (24.83 ± 4.37 kg/m^2^ vs. 27.2 ± 6.05 kg/m^2^; *p* = 0.02), a lower uric acid (6.92 ± 1.87 mg/dL vs. 7.93 ± 1.7 mg/dL; *p* = 0.001), higher PICP (502.6 ± 204.4 µg/L vs. 425 ± 258.8 µg/L; *p* = 0.003) and P3NP levels (244.1 ± 172.9 µg/L vs. 240.6 ± 218.1 µg/L; *p* = 0.0001), a higher LAVI (48.79 ± 15.7 mL/m^2^ vs. 43.69 ± 12.71 mL/m^2^; *p* = 0.036), and a lower GLS (−9 ± 4.8% vs. −10.8 ± 5.6%; *p* = 0.005). The differences in creatinine, hemoglobin, mean E/e’, and ejection fraction did not reach statistical significance.

### 3.3. Primary Outcome: PICP as a Predictor of All-Cause Mortality

**Figure 1:**
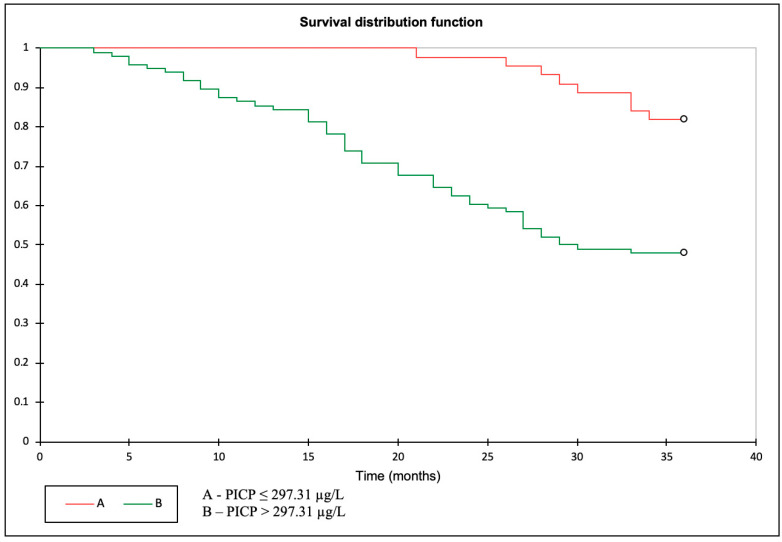
Kaplan–Meier curve displaying the impact of PICP levels on all-cause mortality.

The cut-off point for PICP as a predictor of mortality was established using the ROC curve, at 297.31 µg/L. After dividing the study sample into two groups according to this cut-off, Kaplan–Meier analysis showed that all-cause mortality was significantly higher in patients with PICP > 297.31 µg/L (HR 5.071; 95% CI 1.935–13.29; *p* = 0.001; log rank < 0.0001) (Figure 1).

### 3.4. Primary Outcome: P3NP as a Predictor of All-Cause Mortality

**Figure 2:**
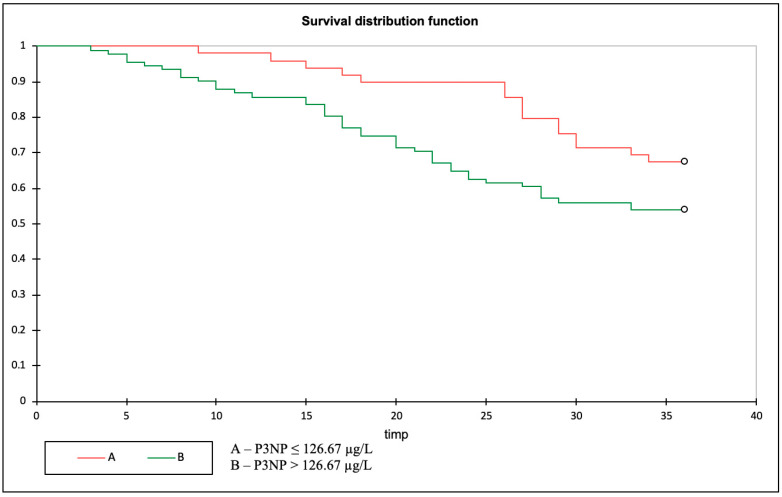
Kaplan–Meier curve displaying the impact of P3NP levels on all-cause mortality.

The cut-off point for P3NP as a predictor of mortality was established using the ROC curve, at 126.67 µg/L. After dividing the study sample into two groups according to this cut-off, Kaplan–Meier analysis showed that all-cause mortality was significantly higher in patients with P3NP > 126.67 µg/L (HR 2.089; 95% CI 1.044–4.178; *p* = 0.03; log rank = 0.05) (Figure 2).

### 3.5. Patient Phenotype According to PICP and P3NP Levels

Table 2 depicts the characteristics of the patients included in this study, when divided by the cut-offs established for mortality. When compared with patients with a PICP > 297.31 µg/L, patients with a PICP level below this cut-off were younger (56.41 ± 3.16 vs. 60.54 ± 16.17 years), had a higher BMI (27.62 ± 6.42 vs. 26.78 ± 4.87 kg/m^2^), less smoking and diabetes (5 (11.36%) vs. 18 (18.75%); 17 (38.63%) vs. 30 (31.25%), respectively), a higher ejection fraction, and a higher GLS (59.02 ± 5.37% vs. 51.16 ± 7.97%; −16% ± 1.9% vs. −8% ± 0.5%, respectively). Regarding P3NP, patients with a value < 126.67 µg/L were older (60.25 ± 12.29 vs. 58.65 ± 16.94 years), had a higher BMI (27.54 ± 6.27 vs. 26.75 ± 4.83 kg/m^2^), less smoking (3 (5.7%) vs. 20 (22.72%)), a higher ejection fraction, and a higher GLS (56.85 ± 5.97% vs. 51.74 ± 8.63%; −13% ± 3.9% vs. −8% ± 0.5%, respectively).

### 3.6. Univariate Analysis and Multivariate Cox Proportional Hazards Model

In univariate analysis, the following variables were associated with all-cause mortality: age, diabetes, PICP > 297.31 µg/L, and P3NP > 126.67 µg/L. A higher BMI and higher uric acid levels were found to be associated with a lower all-cause mortality (Table 3). In multivariate analysis, using a multivariate Cox proportional hazards model, age, diabetes, and PICP above the cut-off level remained independent predictors of mortality. BMI and uric acid were not included in the multivariate analysis, given their protective value against mortality.

## 4. Discussion

This study showed that PICP is an independent predictor of mortality in patients with ESRD who are not yet on dialysis. In contrast, P3NP levels were associated with survival only in the univariate analysis. Additionally, diabetes was confirmed as the most powerful independent predictor of mortality in this population.

Type I collagen is responsible for almost 90% of the bone matrix, being the most important type of collagen in the human body. Synthetized by the fibroblasts, procollagen type I undergoes the action of carboxy-propeptidase, which leads to the formation of collagen type I and PICP, a heterotrimeric glycoprotein. Alongside ‘classic’ cardiovascular biomarkers such as natriuretic peptides or highly sensitive C reactive protein, PICP was proven to have an important diagnostic and prognostic value in heart failure, predicting both cardiovascular and all-cause mortality [25]. The normal range for PICP in the general population is 50–350 µg/L [26]. In patients with an acute myocardial infarction, an early percutaneous intervention leads to a reduction in PICP, while a high serum level predicts left ventricular remodeling [27]. These findings are of paramount importance and contribute to the idea that collagen type I pathway is vital in cardiac fibrosis, especially as the presence of PICP on endomyocardial biopsy is associated with an activation of the fibroblasts [16]. In CKD, myocardial fibrosis is frequently present, representing one of the factors involved in the pathophysiology of heart failure with a preserved ejection fraction (HFpEF) [28].

Data regarding the role of PICP as a biomarker in CKD are scarce, yet promising. Ravassa et al. studied hypertensive patients with HFpEF and CKD, confirming that myocardial fibrosis could be accelerated by the presence of CKD. Moreover, this comorbidity was the only one independently associated with an increased E/e’ and the presence of a more severe diastolic dysfunction; these patients could benefit more from a diuretic treatment [17]. In advanced CKD, especially in patients undergoing maintenance haemodialysis, these mechanisms are exacerbated and PICP preserves its correlation with the severity of the diastolic dysfunction and increased left ventricular filling pressures [18]. The AURORA trial [19], which focused on studying the effect of rosuvastatin in patients undergoing maintenance haemodialysis, included an analysis on PICP as a predictor of CV death and all-cause mortality. The results were promising, showing that an increased concentration of PICP is correlated with a high mortality risk, of similar magnitude as that of hs-CRP. Our study aimed at investigating the prognostic value for mortality of this biomarker in advanced CKD, showing that PICP is an independent predictor of mortality in this population in multivariate analysis (HRa 1.22; 95% CI 1.1–1.31; *p* < 0.0001). This finding could be attributed to a higher level of cardiac fibrosis in this hypertensive cohort, with a mean E/e’ of 9.8 ± 4.3 in the ‘grey’ zone and a preserved mean ejection fraction (53.63 ± 8), but with a significant subclinical dysfunction, as shown by a decreased global longitudinal strain (−10.2 ± 5.3%). Diabetes could influence and accelerate this process, given the high proportion of diabetic patients in our cohort who died at three years of follow-up.

P3NP results from the degradation of procollagen type III by amino-peptidase, in the pathway of collagen type III synthesis. The normal range in the general population is 1.2–4.2 µg/L [29]. This peptide was shown to be associated with organ fibrosis in the liver, lungs, and heart [30,31,32]. Additionally, it was shown that those with higher levels of P3NP exhibit physical deconditioning and loss of muscle strength [33]. In older people, especially those with increased high sensitivity C reactive protein levels, P3NP was shown to predict all-cause mortality, particularly of a respiratory cause [32]. In patients with heart failure, P3NP was proposed as a part of a ‘multimarker’ approach, owing to consistent data regarding its role in the diagnosis and prognosis of this disease [34]. In the RALES trial, a higher P3NP level at baseline predicted the risk of hospitalization and death, and treatment with spironolactone significantly reduced P3NP levels after six months [35]. These findings were later confirmed in hypertensive patients as well, for which P3NP can predict its development and help select those who could benefit from treatment with spironolactone [36]. Data regarding the role of P3NP in CKD are limited to results from the Framingham study, where this biomarker did not predict the development of renal disease [20]. On the other hand, urinary P3NP was studied by Fakhouri et al. as a potential indicator of kidney fibrosis, which may prevent unnecessary kidney biopsies in some individuals [21]. Our study is the first to study the predictive value on mortality in advanced CKD. Patients who died at three years had significantly higher values of P3NP (244.1 ± 172.9 µg/L vs. 240.6 ± 218.1 µg/L; *p* = 0.0001). This could be explained by a poorer physical condition among the deceased. This biomarker performed well in univariate analysis, with a cut-off value of >126.67 µg/L for the prediction of mortality (HR 2.089, 95% CI 1.044–4.178, *p* = 0.03). Still, in multivariate analysis, the P3NP variation lost its statistical significance, probably because of a significant impact of the presence of diabetes in this setting (HRa 1.03, 95% CI 1.021–1.04, *p* = 0.06).

Apart from the data on biomarkers, we confirmed the essential role that diabetes mellitus plays in patients with CKD. In this cohort, diabetes was the strongest independent predictor of mortality, increasing the risk of death up to five times when compared with the non-diabetic population (HRa 5.168; 95% CI 2.590–10.310, *p* < 0.0001). Diabetic kidney disease is the most common complication of diabetes mellitus and is responsible for up to 30–50% of the ESRD cases worldwide [37]. In the setting of diabetic nephropathy, renal fibrosis is essential, but cardiac fibrosis occurs simultaneously. In this milieu, fibrosis is promoted through several mechanisms, such as accumulation of AGEs, TGF-β dependent pathways, activation of renin-angiotensin system, and the dysregulation of microRNAs. Recently, the latter mechanism has been proposed, with several microRNAs being up-regulated in the diabetic heart, thus promoting cardiac fibrosis [38]. 

Another interesting finding was that patients with a higher BMI had a lower mortality at three years, confirming the ‘obesity paradox’ in advanced CKD (HR 0.9; 95% CI 0.838–0.967; *p* = 0.004) [39]. There are several possible explanations for this ‘reverse epidemiology’. Firstly, inflammatory cytokines such as interleukin-6 promote the waste of protein-energy in ESRD, leading to the suppression of appetite and muscle loss. This phenomenon explains the attenuation of the ‘obesity paradox’ in patients undergoing peritoneal dialysis when compared with haemodialysis. Another possible explanation is that the harmful consequences of obesity in the long term are outweighed by the severe prognosis of these patients in the short term. Additionally, the sequestration of uremic toxins in the adipocytes and the interaction between the endotoxins and lipoproteins have been postulated as possible explanations [40].

In our study, elevated uric acid levels exhibited a protective effect on mortality (HR 0.82; 95% CI 0.7–0.97; *p* = 0.02). This finding is not concordant with the existing data, with several papers confirming that hyperuricemia leads to an increased risk of cardiovascular death in patients with CKD and could be explained by the fact that many patients were already treated with allopurinol [41,42]. Additionally, there are data showing that the deposition of uric acid crystals could explain low levels of uric acid in some individuals [43]. On the other hand, Kim et al. proved that elevated uric acid is protective against the development of metabolic syndrome, which could be explained by a pro-oxidant/antioxidant paradox [44]. This paradox implies that an increase in uric acid could be an attempted protective response and that, despite a pro-oxidative effect within the cell, uric acid may have an antioxidant effect within the plasma [45].

Our study faces several limitations. First of all, the design of the study was not a randomized one, with no control group. Additionally, the high mortality recorded at three years (41%), although common in advanced CKD, could still point towards a selection bias. PICP and P3NP are only indicators of organ fibrosis and their serum values would ideally need validation with tissue biopsies, in large, randomized studies. Plasma levels of these biomarkers were measured at a single point in time, from frozen samples, rather than fresh ones, which could influence the results.

## 5. Conclusions

In conclusion, the present study evaluated the predictive value of PICP and P3NP for mortality in asymptomatic ESRD patients who are not yet on dialysis. Our research points towards PICP being an independent predictor of mortality in this special population, which could potentially be incorporated into the actual risk assessment tools, for a better assessment of cardiovascular prognostic. Further prospective, randomized studies could be developed using our findings as a starting point, in order to establish a specific cut-off and other potential implications of these biomarkers in the clinical setting. Additionally, we showed that diabetes is the most important predictor of mortality in this population, confirming that the coexistence of diabetes and CKD determines a synergic increase in the risk of death.

## Figures and Tables

**Table 1:** Baseline characteristics and comparison between the patients who survived and died at three years.

	Study Population (*N* = 140)	Survivors (*N* = 77)	Deceased (*N* = 58)	*p*-Value *
**Age (Average ± SD)**	**59 ± 15**	**53.4 ± 15.9**	**67.47 ± 9.73**	**<0.0001**
Sex (Number, %)	62 F (44.3%), 78 M (55.7%)	38 F, 39 M	20 F, 38 M	0.114
eGFR (Average ± SD) (ml/min/1.73 m^2^)	8.7 ± 3.3	8.29 ± 3.27	9.23 ± 3.41	0.099
Creatinine (Average ± SD) (mg/dL, umol/L)	6.6 ± 2.4 (583.57 ± 212.21)	6.95 ± 2.41	6.19 ± 2.24	0.06
**BMI (Average ± SD) (kg/m^2^**)	**26.04 ± 5**	**27.2 ± 6.05**	**24.83 ± 4.37**	**0.02**
Obesity (Number, %)	36 (25.7%)	22	14	0.56
Smoking (Number, %)	23 (16.4%)	11	12	0.361
**HTN grade (Number, %)**	**11 Grade 2 (7.8%), 129 Grade 3 (92.2%**)	**10 Grade 2 (13%), 67 Grade 3 (87%)**	**1 Grade 2 (1.15%), 57 Grade 3 (98.85%)**	**0.01**
Systolic blood pressure (Mean ± SD) (mmHg)	140 ± 15	137.6 ± 10.6	142.5 ± 13.2	0.06
Diastolic blood pressure (Average ± SD) (mmHg)	76 ± 5	74.3 ± 4	76.7 ± 6.5	0.1
**NYHA class (Number, %)**	**Class I (52.1%), Class II (47.9%)**	**46 Class I (60%), 31 Class II (40%)**	**24 Class I (41.3%), 34 Class II (58.7%)**	**0.038**
**Diabetes mellitus (Number, %)**	**47 (33.5%)**	**15**	**31**	**<0.0001**
Ischemic heart disease (Number, %)	16 (11.4%)	10	6	0.79
Hb (Average ± SD) (g/dL)	9.72 ± 2	9.96 ± 1.98	9.38 ± 1.55	0.06
**Uric acid (Average ± SD) (mg/dL)**	**7.51 ± 2**	**7.93 ± 1.7**	**6.92 ± 1.87**	**0.001**
**PICP (Mean ± SD) (µg/L)**	**457.2 ± 240**	**425 ± 258.8**	**502.6 ± 204.4**	**0.003**
**P3NP (Mean ± SD) (µg/L)**	**242 ± 199.9**	**240.6 ± 218.1**	**244.1 ± 172.9**	**0.0001**
**LAVI (Mean ± SD) (ml/m^2^)**	**45.8 ± 14.2**	**43.69 ± 12.71**	**48.79 ± 15.7**	**0.036**
Mean E/e’ (Mean ± SD)	9.8 ± 4.3	9.64 ± 4.15	10.13 ± 4.61	0.5
Ejection fraction (Mean ± SD) (%)	53.63 ± 8	54.48 ± 7.91	52.45 ± 8.32	0.146
**GLS (Mean ± SD) (%)**	**−10.2 ± 5.3**	**−10.8 ± 5.6**	**−9 ± 4.8**	**0.005**

SD: standard deviation; BMI: body mass index; HTN: hypertension; NYHA: New York Heart Association; * determined by Fisher’s exact test for categorical variables and by independent *t*-test for continuous variables.

**Table 2:** Patient characteristics according to PICP and P3NP levels.

	PICP < Cut-Off (*N* = 44)	PICP > Cut-Off (*N* = 96)	P3NP < Cut-Off (*N* = 52)	P3NP > Cut Off (*N* = 88)
**Age (Average ± SD)**	56.41 ± 3.16	60.54 ± 16.17	60.25 ± 12.29	58.65 ± 16.94
**Sex (Number, %)**	22 F (50%), 22 M (50%)	40 F (41.66%), 56 M (58.33%)	24 F, 28 B	28 F, 50 B
**BMI (Average ± SD) (kg/m^2^)**	27.62 ± 6.42	26.78 ± 4.87	27.54 ± 6.27	26.75 ± 4.83
**Smoking (Number, %)**	5 (11.36%)	18 (18.75%)	3 (5.7%)	20 (22.72%)
**HTN grade (Number, %)**	1 Grade 2 (2.23%), 43 Grade 3 (97.77%)	10 Grade 2 (10.41%), 86 Grade 3 (89.58%)	2 Grade 2 (3.84%), 50 Grade 3 (96.15%)	9 Grade 2 (10.22%), 79 Grade 3 (89.77%)
**Diabetes mellitus (Number, %)**	17 (38.63%)	30 (31.25%)	20 (38.46%)	27 (30.68%)
**Mean E/e’ (Mean ± SD)**	8.17 ± 2.2	10.61 ± 4.83	8.94 ± 3.12	8.92 ± 3.46
**Ejection fraction (Mean ± SD) (%)**	59.02 ± 5.37	51.16 ± 7.97	56.85 ± 5.97	51.74 ± 8.63
**GLS (Mean ± SD) (%)**	−16% ± 1.9%	−7.5% ± 4%	−13% ± 3.9%	−8% ± 0.5%

SD: standard deviation; BMI: body mass index; HTN: hypertension.

**Table 3:** Univariate analysis and multivariate Cox proportional hazards model.

		Univariate Analysis			Multivariate Cox Analysis	
**Parameters**	HR	95% CI	*p*	HRa	95% CI	*p*
**Age, years**	1.147	1.122–1.171	0.001	1.25	1.23–1.28	<0.001
**BMI, kg/m^2^**	0.9	0.838–0.967	0.004			
**Diabetes**	7.291	3.568–11.016	<0.0001	5.168	2.590–10.310	<0.0001
**PICP > 297.31 µg/L**	5.071	1.935–13.29	0.001	1.22	1.1–1.31	<0.0001
**P3NP > 126.67 µg/L**	2.089	1.044–4.178	0.03	1.03	1.021–1.04	0.06
**Uric acid, mg/dL**	0.82	0.7–0.97	0.02			

CI: confidence interval; HR: hazard ratio; HRa: hazard ratio adjusted; PICP: procollagen type I carboxy-terminal propeptide; P3NP: procollagen type III N-terminal peptide; BMI: body mass index.

## Data Availability

Data supporting this study are not publicly available due to ethical restrictions. Please contact the corresponding author for further information.
